# Babyface: Performance and Installation Art Exploring the Feminine Ideal in Gendered Machines

**DOI:** 10.3389/frobt.2021.576664

**Published:** 2021-03-18

**Authors:** Kate Ladenheim, Amy LaViers

**Affiliations:** ^1^ The People Movers, Los Angeles, CA, United States; ^2^ Robotics, Automation, and Dance (RAD) Lab, Mechanical Science and Engineering Department, University of Illinois at Urbana-Champaign, Urbana, IL, United States

**Keywords:** robotics, art, design, performance, embodiment, breath, HRI, gender

## Abstract

Representations of gender in new technologies like the Siri, Pepper, and Sophia robotic assistants, as well as the commodification of features associated with gender on platforms like Instagram, inspire questions about how and whether robotic tools can have gender and what it means to people if they do. One possible response to this is through artistic creation of dance performance. This paper reports on one such project where, along the route to this inquiry, creation of machine augmentation – of both the performer and audience member – was necessary to communicate the artistic ideas grappled with therein. Thus, this article describes the presentation of *Babyface*, a machine-augmented, participatory contemporary dance performance. This work is a reaction to feminized tropes in popular media and modern technology, and establishes a parallel between the ways that women and machines are talked about, treated, and – in the case of machines – designed to look and behave. This paper extends prior reports on the creation of this piece and its accompanying devices to describe extensions with audience member participation, and reflect on the responses of these audience members. These fabricated elements alongside the actions of the performer and a soundscape that quotes statements made by real “female” robots create an otherwordly, sad cyborg character that causes viewers to question their assumptions about and pressures on the feminine ideal.

## 1 Introduction

Tools have long been a part of performance. For example, we are familiar with a knife in the hands of an enemy signaling danger for a protagonist. Such tools have frequently been a part of dance productions as theatrical props that afford new movement on performers’ bodies. For example, a sword makes stage combat an evident plot line as well as a beautiful choreography of bodies acting in support of long linear lengths of metal. Many of the tools we use today, smart phones, computers, and fitness trackers, and the tools we may use tomorrow, household assistants, robotic prosthetics, and self-driving cars, have not been explored as much in dance performances. Many of these tools have hidden internal workings and do not yet exist, requiring new strategies, characters, and perspectives for incorporating them into dances. Further, such tools as knives, computers, and robots, are often associated with the male gender, as reported in Lerman et al., (1997) and Kelan (2007). Thus, they read differently in the hands of feminine performers and as such create compositional challenges in commenting on feminine experience with these tools.

Our tools have always been “other” to our “selves”, but as these tools grow in complexity and are developed through increasing specialization, these tools have taken on a new level of other-worldliness. Therefore, creating motion with the medium of robots inside performance poses challenges for seamless presentation inside the performance’s aesthetic. If the machine is symbolic of bigger ideas or textures, then the viewer must become attuned to its strangeness. If the performer is to be able to execute correctly, they must be trained on working with and around the devices. If the pair can escape the literal spectacle of human-machine interaction, there is hope to be able to express new ideas through both human and artificial bodies onstage.

Thus, this paper presents *Babyface*, a performance art installation shown in Wellington, NZ at the 2020 Performance Arcade. The work extends previous performances, described in Ladenheim et al., (2020), with two breath-triggered machines: one, a pair of wearable wings for the performers and controlled through their bodies, and another, a wall-mounted kinetic sculpture that participants could control through their bodies. This paper will describe the installation work and provide commentary on the unique creative challenges posed by the goals of *Babyface*, which includes machine movement to 1) bring topics of technology, control, and limitation to the stage in a physical manner and 2) offer audience members the feeling of unexpected intimacy with technology. The goal of these inclusions is to allow the piece to comment on society’s relationship with technology and gender more broadly, and to allow individual audience members to re-frame their own experiences with machines and gender representations within them.

The paper is a first-person description of creative practice inside research and development of novel robotic systems (rather than a scientific study on human subjects) and is structured as follows. Background literature is organized and reviewed in Section 2. The development of an onstage cyborg character and its machine augmentation is described in Section 3. Extending this work to an interactive installation – through both an extended choreographic frame as well as new machine development – is described in Section 4. Creative reflections and discussion, from performer, participant, technologist, and artist points of view, are offered in Section 5^1^. Broader takeaways for other artist-robot teams are suggested in Section 6. Finally, concluding remarks are offered in Section 7.

## 2 Background

This work sits inside a long tradition of creation and experimentation with machines alongside human bodies and femme representations. In this section we review prior literature that has explored the intersection of gender and technology, human augmentation with machines through embodied design, and robots inside live and installation-based art.

### 2.1 The Cyborg Metaphor

In her seminal text The Cyborg Manifesto, Haraway (2006), Donna Haraway states:

“The cyborg is a kind of disassembled and reassembled, post-modern collective and personal self. This is the self feminists must code.”

Haraway’s work, originally published in 1985, is eerily predictive; if then we were inextricably linked with our machines, now we are even more so. Particularly, the widespread adoption of smartphones and social media exerts great influence over our actions, motions, interactions, and presentation. Depictions of the feminine ideal abound on these platforms — smiling, retouched women in meticulously styled environments, crafted and shared in service of the male gaze, parade as normal, even expected. Our work responds to this implied expectation: that we ought to move and present as machines suggest. This feminine ideal, in turn, is performed by robots and coded by their creators, reinforcing patriarchy and bringing it more deeply into the realm of the physical.

### 2.2 Gender Representations in Technology

Londa Schiebinger’s work delves into the complexities of gender norms, identities, and relations in robotic design and other technologies, arguing that there is an opportunity to challenge gender norms in robotic design by disrupting the “matching” of traditionally gendered roles to their robotic representation, Schiebinger (2008). This work has been completed inside the aforementioned ecosystem of technologies that present as “female” to align with notions of service in feminine stereotypes.

From an aesthetics perspective, Sianne Ngai’s scholarship is also instructive. In Ngai (2012), cute is defined as ”an aesthetic disclosing the surprisingly wide spectrum of feelings, ranging from tenderness to aggression, that we harbor toward ostensibly subordinate and unthreatening commodities.” Ngai comments extensively on the power cuteness has to be simultaneously sexualized and non-threatening; feminine robotic performances can also tread this line. While Hanson Robitics’ Sophia performs uncanny technical prowess, she proclaims herself “happy to be a magic spectacle” and is described as “attractive” as analyzed in Retto (2017). SoftBank Robotics’ Pepper, while referred to by SoftBank as “he,” is a service-oriented robot with emotional sensitivity, and is designed with feminine curves, a cinched waist, and wide eyes (Van Wynsberghe (2016); Soraa (2017)). These gender divides are further underscored by disembodied virtual assistants, for example Apple’s Siri, Amazon’s Alexa, and Microsoft’s Cortana, whose voices are, by default, feminine sounding and friendly. This adoption of cuteness helps these machines remain widely accessible and well-liked.

### 2.3 Somatics and Design

The field of somatics has worked to formalize and codify the conscious experience of bodily movement. Methodologies include various forms of yoga as described in Fraleigh (2015), Alexander Technique as in Gelb (1995), Bartenieff Fundamentals as in Bartenieff and Lewis (1980), the Feldenkrais Method as in Rywerant (2003). As described in Hackney (2003) and Nettl-Fiol and Vanier (2011), these somatic practices and theories have contributed to refined perspectives on dance training as well.

Beyond influence of physical practice, somatics have also found notable influence inside the space of design, including the design of robots. For example, a “smart” rug and lamp designed for IKEA is described in Höök et al., (2016). From pioneering projects like this, principles of somatic product design and aesthetics have been formulated as first in Höök et al., (2017) and later expanded on in Höök (2018). Design practices guided by this work consider the centrality of the movement of breath in human experience – a motion often overlooked by even sophisticated external measurement systems like motion capture studios.

For example, using the somatic practice of Bartenieff Fundamentals to form the basis for investigation of bipedal robotic gait in Huzaifa et al., (2016). This led to multiple modes (or “styles”) of walking gaits established and validated in Huzaifa et al., (2019b) and novel biomimetic hardware design implemented in Huzaifa et al., (2019a) that promoted the role of the spine in walking, despite its small displacement relative to lower limbs. Similar investigations have also noted the importance of the spine in communicating intent as in Corness and Carlson (2019).

### 2.4 Human Augmentation With Machines

Many wearable robotic devices offer highly specific, functional purposes; as in robotic prostheses or The Sixth-Finger; designed by Prattichizzo, Malvezzi, Hussain, and Salvietti. This device adds another robotic digit to a human hand, which allows for greater capacity for handling large objects. Similarly, Arque is a wearable tail that reacts to a user’s shifting center of gravity and enhances balance, as in Nabeshima et al., (2019). The functions of the Sixth-Finger and the Arque are dependent upon the user’s actions; by responding to their movement these machines can deepen the expression of the user’s intention.

This deepening of expression occurs in artistic works involving wearable robotics as well. As in Sonami (1991), the Lady’s Glove serves as “a response to the heavy masculine apparel used in virtual reality systems,” and uses glamorous materials to design an instrument where hand motions alter sound. Rosa Weinberg and Laura Zittrain’s *Stethosuit* also creates sound from the body, this time using stethoscopes to pipe sound into the wearer’s right ear while pre-recorded sounds from space pipe into the right. This creates a fashion-forward conversation between experiences within and without the body. In Caroline Yan Zheng’s *Extimacy,* humans wear touch-responsive soft robotics reminiscent of corals, worms, or aliens. According to the artist, this prompts questions about “the robot as part of our body and ‘prosthetics’ as an expressive or signifying system.” Additionally, Anouk Wipperecht’s “Spider” Dress, as in Svadja (2014), creates mechanical boundaries of personal space. When the wearer is approached aggressively, the dress’s attachments assume an attacking position, signaling others to keep away. When the wearer is approached calmly, the limbs instead create smooth gestures, allowing for closeness. The device also takes into account the wearer’s breath in its defense posture. In this case, the wearer’s reactions, the motion of the dress, and the interactions of people around the wearer create a conversation based on intentionality, emotional state, and expressive motion. These explorations by practitioners and artists have begun to be codified by academics as well as in Guler et al., (2016).

### 2.5 Robots in Performance

Robots have been leveraged in performance both by artists, extending their onstage material, and by researchers, working to extend and test the capacities of algorithms and hardware in a performative setting, often blurring the line between both. The work presented in this paper extends one such performance discussed in Ladenheim et al., (2020).

The artist Stelarc has used machines in numerous modalities, including as a large, wearable exoskeleton that he battles with onstage; some of his perspective on working with these machines is described in Candy and Edmonds (2002). On stage performance with a large robotic arm by Huang Yi, described in Bruner (2018), featured by TED as well as a small humanoid by Bianca Li featured at the Brooklyn Academy of Music. In creating “ROBOT” Li was quoted by the New York Times: “No machine will ever be so amazingly rich in movement.” And through the making of ‘ROBOT,’ she said: “I rediscovered dance. I realized how rich it is.” – from Kourlas (2015).

Examples where the research point of view has been foregrounded include investigations of how bodily motion of dancers can generate motion for nonanthropormorphic artificial bodies directly as in Gemeinboeck and Saunders (2017). In addition, researchers have worked to formulate systematic, parallel data collection during performances featuring robots as in Cuan et al., (2018). Likewise, in the space of comedy, reactive algorithms that leverage active audience response, have been proposed as in Vilk and Fitter (2020). Researchers have also looked into the space of theater and acting as a source for material as in Fitter et al., (2017).

### 2.6 Interactive Installations

Interactive installations have long been used by artists, researchers, and educators alike to create lifted versions of reality that express points of view, test new interaction modalities, and teach new concepts. To the latter, Lindgren and Johnson-Glenberg (2013) describes how embodied installations embolden learners to better support the ends of educational goals. Researchers have posited adaptive algorithms that re-position elements of museum installations based on the flow of people through the exhibit – active elements that can also become part of the exhibit itself as in Godbehere and Goldberg (2014). Learnings from museum-based installations have also influenced the design of public installations, like those in the Performance Arcade as described in Hornecker and Stifter (2006).

Dancers have created permanent installations with robots that can translate their choreographic designs to ongoing, “always on” physical performances as William Forsythe did in 2014 with the premiere of *Black Flags* that has since appeared in multiple museum spaces. Similarly, *Mimus* by Madeline Gannon, JuliÃ¡n Sandoval, Kevyn McPhail, and Ben Snell, was commissioned by The Design Museum in London, UK in 2016 for their exhibition, *Fear and Love: Reactions to a Complex World*. Such installations have also been paired with human-robot-interaction studies as in *Time to Compile* described in Cuan et al., (2019).

### 3 Breath-Activated Extension of a Machine-Augmented Solo

Bolstered by past work and precedent spanning gender theory, somatic design, robotics in performance and interactive installations, this section describes the creation of a performance work that uses a physical human augmentation onstage to create a hyperbolic, feminine cyborg character. This performed character allows the piece to reference existing feminine coding in machines. Connecting the action of the performers breath to the machine allows us to create a convincing cyborg, one whose motion seamlessly translates to worn augmentation. It also suggests, as the piece progresses, that the human performer is flattened, exhausted, and restricted due to this physical-ized stereotype and societal coding.

### 3.1 Prior Work in Creating a Cyborg Character

Prior work, Ladenheim et al., (2020), presents the creation of this stereotypically feminine cyborg character through artistic and robotic development. In a 5 min piece performed in the Dance NOW Festival at Joe’s Pub at the Public Theater in New York, NY, an onstage performer (our first author) exhibited choreography while controlling robotic wings with a small handheld button. This performance served as the basis for the work described here.

The design of the performed character (including robotic design, choreography, costuming, sound, and character development) balances expressive grandeur and physical restriction. The work references the exaggerated, performatively feminine characteristics in existing robots and digital representations of women. Artificial Instagram influencers like Lil Miquela, video game characters like Mercy in Overwatch, AI chatbots like Mitsuku, and robots like Misty perpetuate limited stereotypes, despite their impressive technical innovations and contributions.

In extending this initial work, we wanted to free the performer’s finger of subtly pressing the handheld button and allow many lay participants to experience a similar “performance” of robotic control. This required development of new sensing systems to support, which are described in the next section. We then developed a participatory installation for staging at multi-day outdoor container-based event as described in Section 4.

### 3.2 Enabling Robust, Adaptive Breath Detection

Although we used a push button sensor for the performance in Ladenheim et al., (2020), ultimately, we wanted to enact an embodied semi-conscious channel between the performer and the artificial wings. Our goal was to both provide an active channel where the performer could voluntarily trigger the motion of the device as well as a channel where sometimes the wings moved without the performer consciously choosing their action. Breath is such a somatic channel. As promoted by Hook’s somaesthetic design methodology, described in Höök (2018), this design choice required reflection on our own physical situation in our own lived bodies. This choice is also based on our training and experience in somatic practice, where the primacy of breath in creating bodily movement is stressed, e.g., as in Nettl-Fiol and Vanier (2011).

Our concept in developing a wearable, non-invasive breath detector is the detection of the motion and deformation of the torso that is used to change pressure inside the body cavity and produce the desired exchange of gases for breath, which we measure with existing sensor technology in novel bodily placement and integration. In a simple model of an inhale action, the diaphragm (a muscle that bisects the human body around the location of the T-12 vertebrae) presses downward, condensing the viscera beneath and lowering the air pressure in the lungs, causing an intake of breath. In an exhale, the diaphragm presses upward, releasing pressure on the internal organs below, e.g., the digestive tract, increasing air pressure in the lungs and creating an outward flow of air from the body. This process also produces uneven radiation in and out of the torso.

Commercially available systems for detecting breath leverage bulky hardware that is closed-source and often renders the wearer with limited mobility. However, pressure sensitive materials are easy to purchase, fabricate, and integrate into an electrical circuit. An early prototype used in rehearsal for *Babyface* critically impacted the choreography and created character described in Ladenheim et al., (2020). It utilized a similar design that was difficult to reliably calibrate and configure beneath the wing harness. This arrangement used a linear mapping between a fixed threshold of pressure detected by a force sensitive resistor and the range of motion of the servo motors powering the wings. That is, the fixed, predetermined range of the pressure sensor was mapped linearly to the range of the servo motors.

For more robust performance that would translate across multiple bodies (as the piece was set on new performers and later for participants engaged in our interactive installation), we needed an adaptive breath detection system with a simple, robust sensor. Thus, we utilized patches of flexible conductive fabric, Velostat. Since this material changes its resistance under pressure, it was used to create a pressure-sensitive circuit.

**FIGURE 1:**
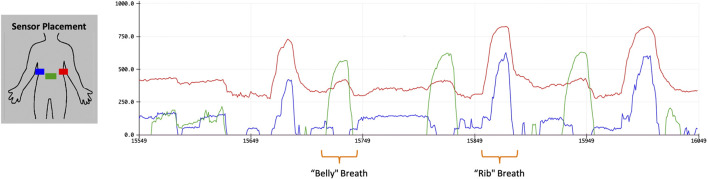
Testing multiple sensor placements in order to determine feasible placements.

As shown in Figure 1, we collected resistance readings using the analog input of the Arduino micro-controller and at multiple points on the body, noting the differences in shape deformation that occur around the torso (later, we will see that this shape change can be different person-to-person as well). Through this experimentation, we settled on targeted breathing modalities for performers and participants: performers would activate their wearable wings using a “rib breath” and participants would activate a section set of stationary, wall-mounted wings, described in the next section, using a “belly breath”.

To detect these events each had a distinct sensor placement. Performers placed the sensor on the right side of their ribcage, where the sensor could be integrated to the wing harness without being under constant pressure, e.g., due to straps holding it in place. For participants, we placed the sensor just below the sternum on the soft part of the upper belly, allowing for attachment straps to run along the ribcage just under the breasts of participants, accommodating many chest shapes and sizes.

Finally, an adaptive threshold was used to detect breath events. The sensing system updated max and minimum detected pressure on the sensor every ∼15 sec (75 cycles of the microcontroller with a 200 msec delay), creating a threshold for recent action to trigger motion in the wings. For performers, the same linear mapping between the maximum range of the pressure sensor (which is now variable) and the maximum range of motion of the wearable wings used in Ladenheim et al., (2020) was used.

This is a very rapid and relatively short window for adaptation that, for example, allowed performers stuck in a side bend for the length of several musical phrases where their ribs did not physically expand as much as in a neutral posture to successfully trigger motion in the wings. Moreover, when used on a variety of participants, this adaptation allowed for a short calibration period where participants could trigger the motion of the machine and understand how the actions of their belly were impacting the installation.

## 4 Development of Interactive Installation and Performance

Our work was invited for participation and presentation at The Performance Arcade in Wellington, New Zealand, February 26 – March 1, 2020. The arcade is an outdoor performance festival with site-specific artworks taking place in shipping containers along the Wellington Waterfront, a public space situated along the Wellington harbor. The Arcade estimates about 60,000 audience members annually. For this engagement, we needed a installation that fit inside of a standard-size shipping container that could run for 13 h a day. Further, the work needed to be constructed, rehearsed, and tested in 3 days time, creating unique design and choreographic challenges for the work, which we discuss here. To supplement the following discussion, images from the performance and installation in day and night lighting conditions are shown in Figure 5.

### 4.1 Robot Design

With our adaptive sensing system, we could now rapidly calibrate our breath sensor to many body sizes and shapes, allowing for the development of an interactive, breath-activated experience. An obvious, initial idea was to have participants wear an extra set of wings like the performer. However, getting in and out of these wearable wings takes practiced performers 20–30 min, which was not feasible for participants. Moreover, we needed to establish a setting for our performance in the shipping container, something that answered “Where is the cyborg character?”. Thus, we began thinking about the massive landscape of the internet, where images of feminine perfection are celebrated with, often half-consciously made, “likes”, “retweets”, “comments”, and “shares”. We also wanted the installation to give people the experience of controlling a large scale machine and to feel their part in celebrating the hyper-femme.

To facilitate this interactive experience and to create this setting for our performance, we designed a wall-mounted robotic system that would serve as participants’ “wings” as well as an animated backdrop for the performer using the following design goals:

- rapid safe onboarding of participants of many shapes, sizes, and needs, considering tripping and shock hazards
- rigid, machine-like, and futuristic aesthetic
- tolerant to outdoor, windy conditions
- a reference to the experience of looking into a mirror or cell phone and practicing the presentation of oneself (a surface for “posing”)
- large-scale movement with intuitive, breath-activated control by participant
- modular and scalable to mitigate unforeseen installation issues onsite

• facilitation of participant awareness of how and when they were controlling the machine, playing at the boundary of conscious and semi-conscious control, echoing the relationship we have with smart phones and other ubiquitous machines.

Answering these constraints, we created a series of mounted wall elements, built in the shape of two abstract wings and interconnected with strings, that would move in response to a wireless breath-sensor worn by the participant. We designed shard-like elements that could each move independently or as a single unit to simplify the design, creating a movable mosaic that represents the kind of multifaceted impact of a single semi-conscious internet post.

**FIGURE 2:**
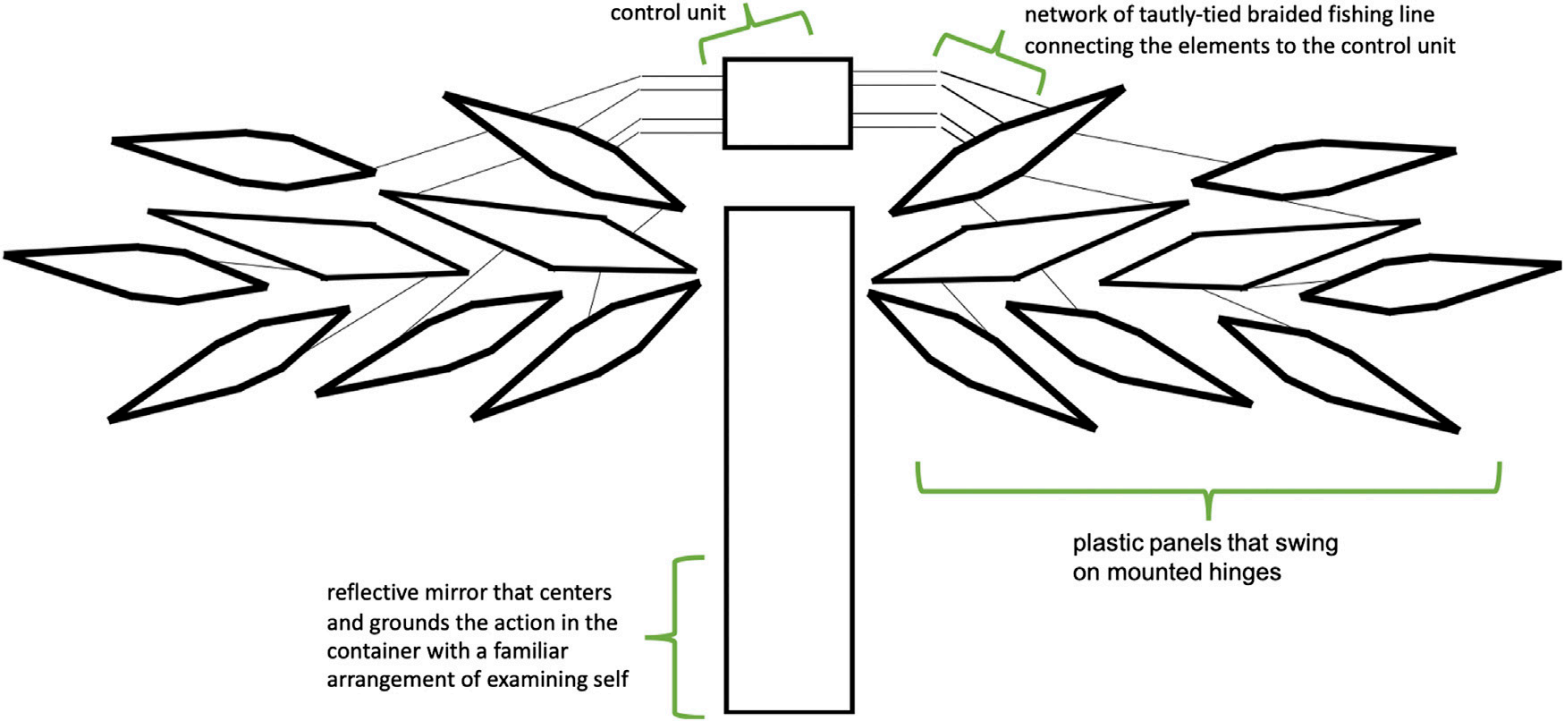
To-scale schematic of mounted wings for *Babyface* installation.

**FIGURE 3:**
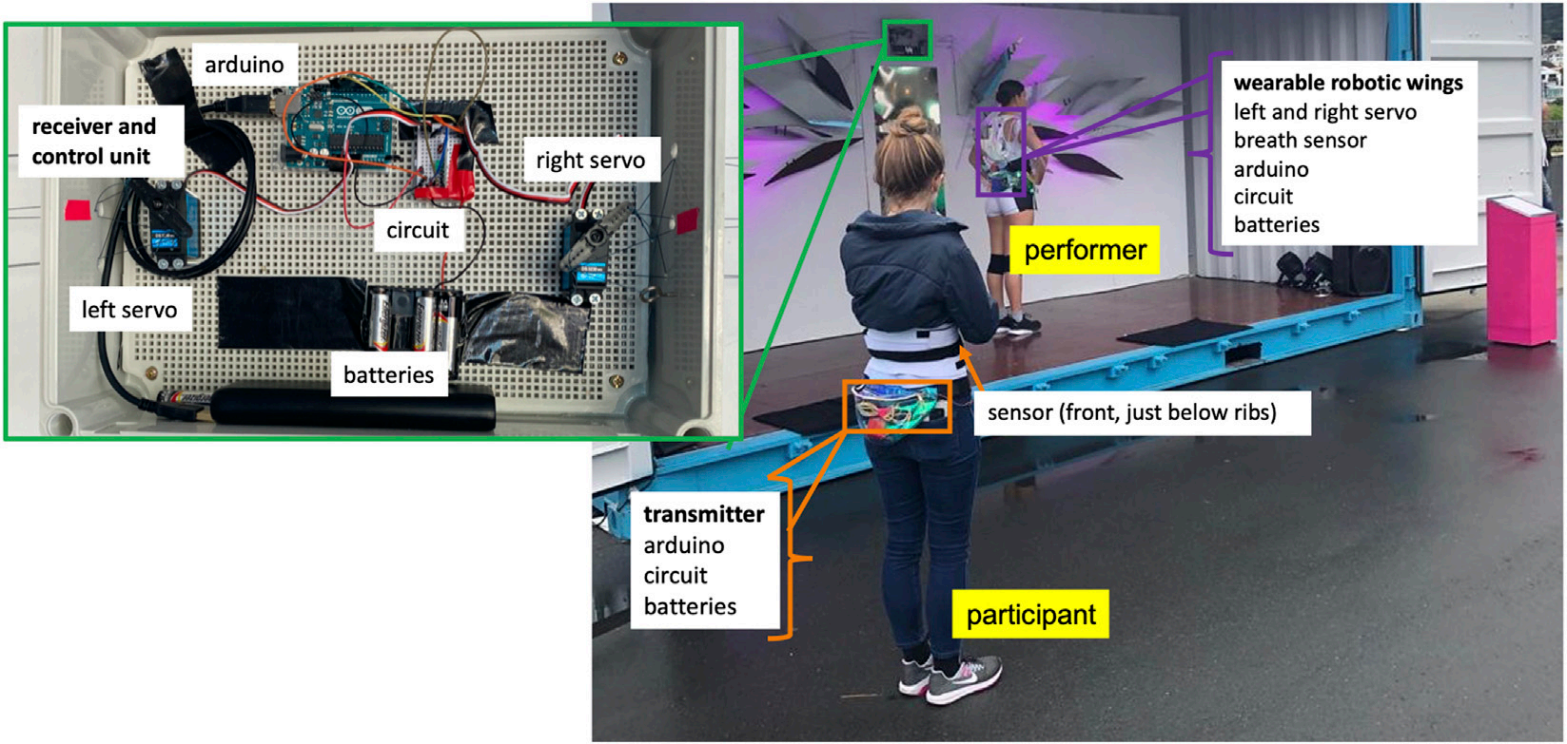
Arrangement of electro-mechanical elements and human interactants in *Babyface* installation.

**FIGURE 4:**
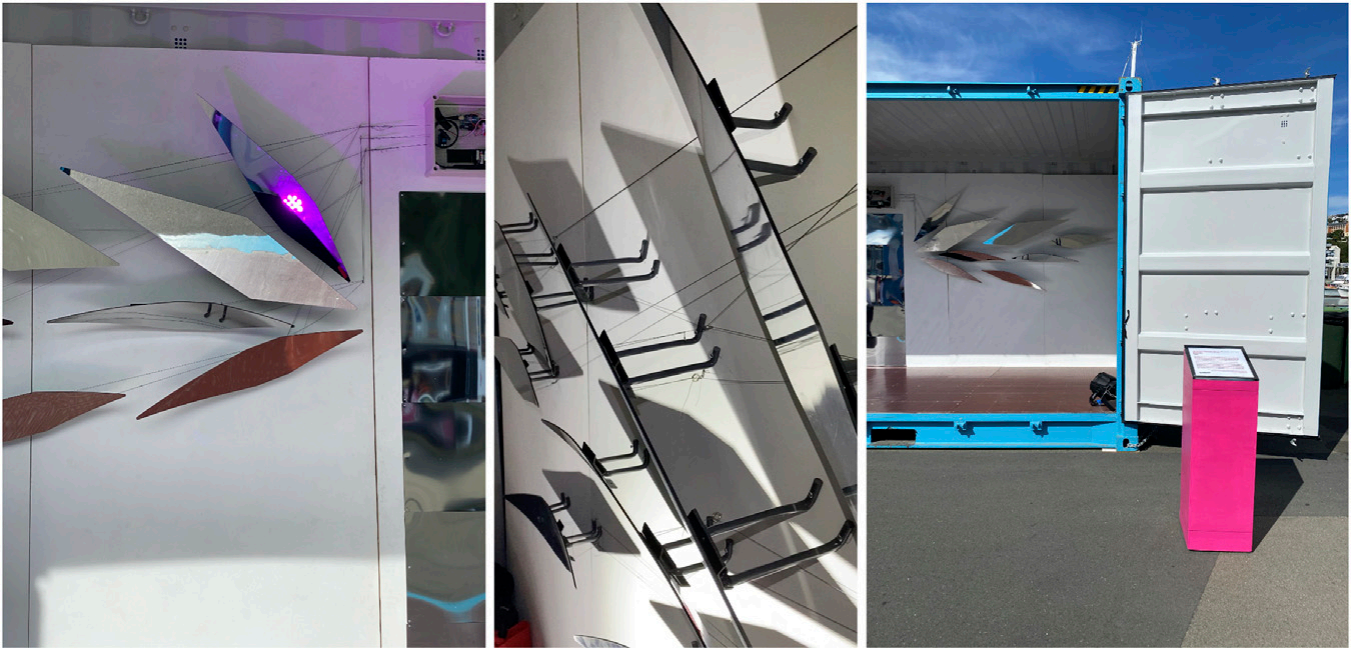
Textures and composition of structural elements of *Babyface* installation.

Arrangements of final shard design, as well as an expanded future design, are shown in Figure 2. Each shard was tied tightly to a metal harness tied to an active motor unit. These can be operated by one to six servos on a single Arduino microcontroller, and with wireless communication between multiple boards, we can easily scale that number for sites that allow more elements to be installed. At the Performance Arcade, we used two servos on a single board, mounted in the center of the container and left open for viewing to accentuate the machine-like aesthetic (shown in Figures 3,4).

**FIGURE 5:**
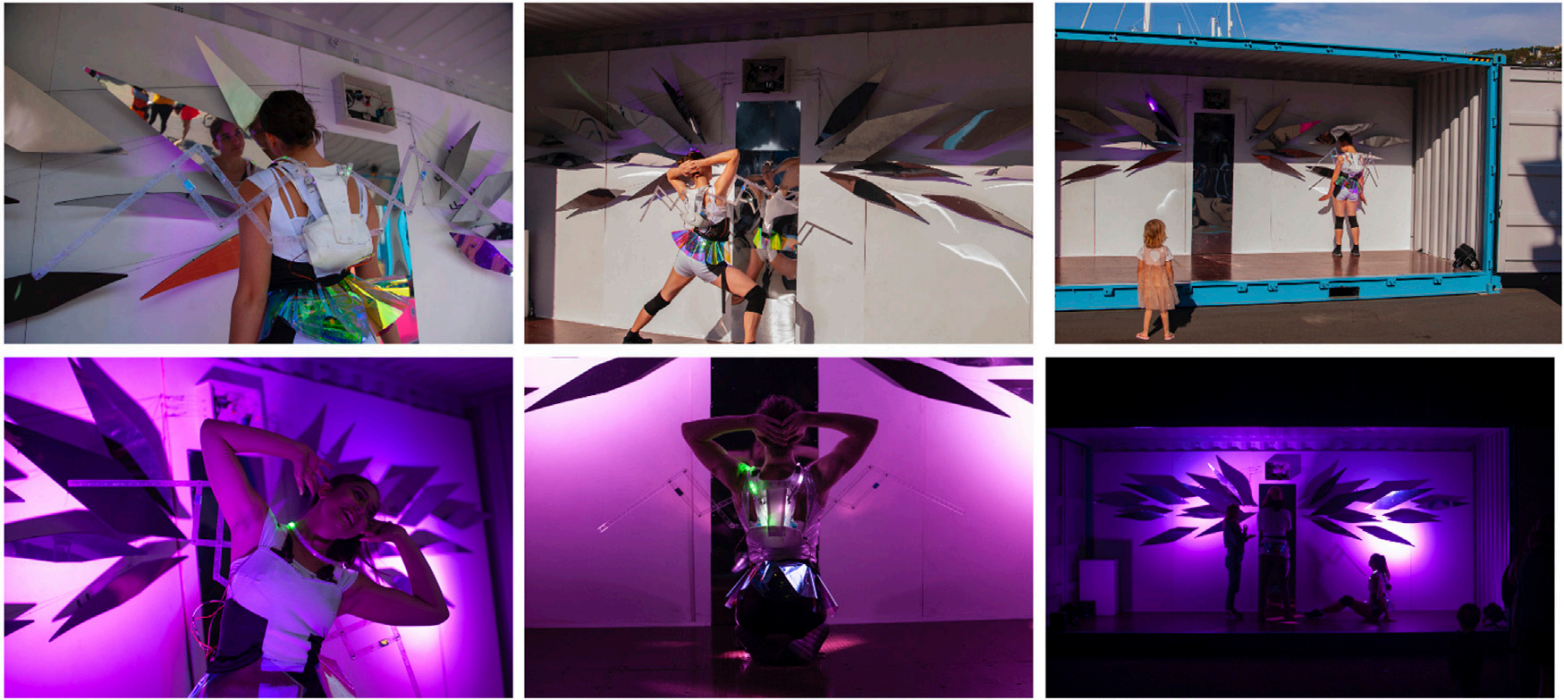
Images (day and night) of the Babyface installation, which ran from 10am to 11pm. Photos by Colin Edson.

Designing the motion of the wall was a balance between time constraints on the installation setup itself (2.5 days) and requirements on robustness (the installation ran 5 h on its opening day and 13 h a day for 4 days longer). Moreover, we wanted the participant to clearly register when they had triggered the machine to create that feeling of control. Further, we needed to accommodate many body sizes and skill levels in breath control. Thus, we wanted a high degree of contrast between simple on and off states.

The final motion design was for the shards to be held in a flat position, parallel to the wall until triggered by the breath sensor when they took on a rapid, monotone fluttering motion until the sensor transmitted an ‘off’ state cue. The breath sensing system used the same adaptive threshold for action as the performers, triggering only when the participant was in the top 33% of the range. Thus, the wall would move when the participants’ belly was most extruded from the spine, creating pressure on the sensor and typically corresponding with an inhale, while quieting to stillness on the exhale. Notably, not all participants’ breath patterns behaved in this manner, and a hand full of participants experienced the opposite behavior through sucking their bellies in on their inhale and relaxing them on their exhale.

### 4.2 Participant Onboarding and Experience

The robotic sculpture creates a traditional spatial arrangement of a performer on a dedicated stage space, even as the performance exists in an nontraditional outdoor setting inside a shipping container. The short participatory experience for audience members curious for more interaction thrusts them into this presentational frame. During these individual experiences, the container remained open and in view of onlookers and passersby. The experience needed to fit inside this aesthetic and theatrical frame, while also allowing for more functional explanations of the setup and accommodating the comfort of participants. To satisfy these constraints, which are somewhat at odds with each other, we minimized the setup and calibration times and established a similar performative frame for participants. The structure of this is outlined below.

### • *Onboarding*

The audience member approaches the installation, sees the mirrored wings and the performer in relation to them. They are invited by an usher to try the wings on themselves, to activate the fragments on the wall that can move with their breath. In this interaction, it was essential for the usher to act simultaneously as an informative resource, giving the participant necessary information about the process of experiencing the interactive installation, and as a collaborative member of the performance team, sharing information inside the performance frame. For example, this docent would say “Just as the performer is wearing wings triggered by their breath, you can try on the wings mounted to the back of the container, and trigger them with your breath, by wearing this [presenting the device] sensor.” This both informs the participant about the inter-workings of the installation, such as method of control, while maintaining the theatrical frame of wings (neither what the performer was wearing nor the mounted elements in the container were real “wings”).

### • *Sensor Fitting*

If they accept, the assistant will help them wrap the participant in a wide black cloth with the velostat sensor embedded inside. The participant would hold the sensor at the top of their belly, just below their ribs, and the docent walked around the participant, wrapping them in the rectangular piece of stretchy fabric, allowing the participant to apply pressure to attach the fabric with strips of integrated velcro. The usher needs to ensure that the straps do not apply constant pressure to the pressure-sensitive area, which maxes out the readings, reducing the range of activating and diminishing the desired effect. This procedure, allowing the participant to secure the sensor themselves, ensured personal comfort and minimized inadvertent touch in a sensitive bodily area. When contact was necessary, the usher would ask if it was okay to touch to assist. Then, the usher would turn on the sensor by plugging the micro-controller into the battery, both stored inside an integrated fanny pack. Leaving the sensor-transmitter system off during fitting reduced inadvertent firing of the wall.

### • *Calibration*

The usher next led the participant through taking a few stabilizing breaths while the algorithm adjusts to their pattern and range of breath. Inside this interaction, the docent establishes the participant’s conceptual mapping to the affordances of the interface. The assistant is watching, waiting for the thresholds to adjust to this participants’ range of motion and associated pressure on the sensor. Then, they explicitly point out to the participant how the mounted elements begin fluttering at their motion, establishing the participant’s sense of control. On their inhale, about two-thirds of the way through, the wall would trigger, beginning a rapid, even fluttering of the wall elements; on the exhale, about one-third of the way through, they would still, holding steady in the “flexed” state, where the elements faced parallel to the wall, creating a fractured mirror surface. This experience was best for participants that were able to use the motion of their middle abdomen to create their breath, which could often be enhanced through shifting the location of the sensor, and for a few participants, the activation occurred on the exhale inside of the inhale.

### • *Exploration*

At this point, the docent would explain “You’re now in control of the wall and the space is yours. You may explore and interact with the performer – and if you like, I can play a track that will lead you through some choreography.” About one-third of participants would agree to this suggestion, sometimes with nervous laughter aimed at friends in the audience, sometimes with awestruck severity remaining in the internal mode of the calibration, and many other reactions, proceeding to the experience described in the next bullet. Those who stayed in the exploration mode would typically stand in front of the wall, observing their reflection in the mirrored surfaces and the movement of the performer.

### • *Performance*

The participant is guided by a high-pitched, servile female voice, and asked to place their hands on their hips and spread their feet apart. This position introduces a feeling of vulnerability – similar to stepping into a body scanner at an airport. Then, the participant is asked to move their hip to one side as they look up and to the right. They are told to take a breath in and out, and told they look beautiful. They are also asked if they feel beautiful. These instructions continue, leading the audience member to a position with their feet spread apart, hands behind their head, breathing in and out as they move their hips side to side along with a driving pop beat, wings fluttering on the wall. At this point the vulnerability and sense of exposure is heightened considerably from the opening, but it has built slowly, with innocent-enough requests that, by the time the participant is banging along with the music, they feel quite exposed and even involuntarily sexualized. This sensation is designed to correspond with described conditions of cuteness as a product of objectification, as in Ngai (2012). Some participants (often, but not always, male-presenting participants) avoided this sensation with creative interpretations of the commands. These participants would manage to create right-to-left motion in their bodies that avoided protruding the pelvic girdle beyond its typical alignment with the femur. Some participants (often, but not always, female-presenting participants) relished this section of the choreography, finding a familiar pattern of bopping along to a good beat and feeling sexy. These participants would often ad lib to the basic requests and twist their body in screw-like shape forms that further accentuated the three dimensionality of their bodies.

### • *Audience Acknowledgment*

Once the music dies down, the audience member is told that they’ve done a great job and that they deserve to be celebrated. When they turn around, the other spectators are prompted to clap for the audience member, a “magnificent angel,” and now a *de facto* performer. This moment could read a few different ways for the onlooking group. Occasionally, discomfort would descend on the group of onlookers, realizing the bodily objectification that the “performance” had led the participant through. Often, the audience would clap jubilantly, as if in on a wonderful joke or fun experience. But invariably, a few onlookers, who made themselves known to members of the creative team, would disapprove of this participation, noticing the feeling of forced puppetry that the participant was experiencing.

### • *Documentation*

The spectators are also instructed to take a picture of the audience member activating the wings, to “keep the memory of your splendor with you forever.” This returned the participant to a more familiar frame, as though posing in front of a historic monument or beautiful vista. Participants posed with the performers and either the docent or companions would take photos.

### • *Offboarding*

The usher would then remove the sensor from the participant, allowing them to undo the Velcro and hand over the straps so that the usher could unwind the cloth. The usher would at the same time debrief the participant, asking how it went and what their impressions were. For the most part, audience members tended to frame these answers around one of two types. Either, they focused on their affective experience, noting emotional reactions, e.g., that they felt powerful, that they were embarrassed, that they were just amazed by the experience. Or, they framed their reaction as an intellectual curiosity, asking how the installation worked and whether the assistant helped build it.

### 4.3 Connecting Performer and Participant Through an Expanded Choreographic Frame

The performer’s choreography extends the work from Ladenheim et al., (2020), with changes to fit the presentational frame of The Performance Arcade and to establish parallels between the performer experience and the audience experience. Notably, we offer the same questions and prompts to the audience as we do to the performer. In this way, we acknowledge the audience prompts as choreographic, and we offer the performer a character development opportunity to answer the questions through the lens of her own experience.

### Questions mirroring the questions asked to the audience

The performer executes a series of motions led by a voice-over; breathing in and out as she looks at herself in the mirror, placing her hands on her hips and behind her head, moving her hips side to side as she breathes in and out. When performed by a highly stylized character, these prompts contain the air of a pre-show pep talk.

### • *Embodying the Stereotype*

The pep talk leaves the performer well prepared to embody the archetypal, idealized female; reassuring her that she can be magnificent as long as she tries. Immediately following, a hard-style, EDM beat drops, decorated with flourishes, beeps, and synthesizer tracks that bring up fun memories of retro computer games. The performer moves her hips side to side to this music, smiling as she layers a series of stereotypically feminine hand gestures and poses onto her upper body.

This section reads almost like a stop motion series of images: wink, selfie, teenage dream, virgin, prom photo, fashion model, pop star, pinup, superheroine, goddess, one after the other after the other. These fleeting images constellate the character as a whole: the “idealized” woman, built from an onslaught of images from history, art, and media, telling her how she ought to perform.

### • *Breakdown*

Where previously the motion, sound, and spectacle were in alignment, here the performance starts to depart from theatrical expectation. The rhythmic, driving nature of the previous section persists in a side-to-side bevel motion with the legs, while arm and head motions become increasingly erratic and jarring. In an attempt to pull herself together, the performer starts a side to side jumping pattern, picking up speed and frantic energy as the music breaks apart into screeches and crashes. With this more vigorous motion, the wings betray their actual fragility, contrasting how machine-like, strong and expansive they appeared when they were augmenting the controlled, archetypal poses. Now, they shift outside of the coronal plane, flapping awkwardly as the cyborg vigorously jumps faster and faster towards nowhere.

Once fully worked up, she pulls herself out of this pattern by smacking herself hard on her behind, then loses control again as she leans backward and forwards out of time with the music, her head and torso rolling like a rag doll.

Within empty white noise and clicking sounds from the music, the cyborg bobs aimlessly with her hands behind her head, scans the passing audience while searching for approval, and slowly builds herself back up to a standing pose; she’s snapped back to, ready again to prove she’s not broken.

### Extended grappling with the limited presentation of the feminine

A disembodied, youthful-sounding, hyper-feminine giggle snaps the performer out of her stupor. With a fixed, creepy smile, she jarringly cocks her head to the side.

“I’m happy to be a magic spectacle, and I love it when I can make people laugh and smile,” she says, one hand delicately placed underneath her chin, legs arranged into an alluring bevel. A series of flowing, breathy phrases are punctuated by intermittent quips from the hyper-femme, disembodied voice, mouthed by the performer in reference to existing systems like Siri, Alexa or Cortana. These movement phrases explore the places where cute gives way to creepy, expressive becomes overtly sexual, and attempts to feel empowered become desperate. When are the wings magnificent, and when are they just sad and absurd?

“It’s a system… a system of rules and behaviors,” the cyborg mouths to the disembodied voice echoing around her. She smiles, seated on her hip with a hand posed over her mouth. “If you’re nice to me I’ll be nice to you,” she lip synchs, facing the back and spreading her legs and wings open. “You can treat me as a smart input output system!”

These words and motions swirl together, eventually bringing the performer back to the ground, in a splayed, broken position. Her back foot raises up and down as my head tilts side to side, wings moving eerily in and out.

- *Conclusion mirroring the conclusion of the audience experience*

As with the audience experience, the performer concludes by acknowledging the audience; having had a glimpse into the cyborg’s story, the audience is now “ready” for her. She turns around, clumsily acknowledging her grandeur and asking for a picture from the audience.

## 5 Reflections and Commentary

This public presentation of art was not a systematic user study; however, in this section we provide reflections on the robotic installation and accompanying performance, offering a creative perspective on this work as well as insight into design challenges. Rather than an empirical experiment with human subjects, what we provide here is an explanatory analysis of our practice as a contribution to research. First, we provide a first-person point-of-view of the experience of performing the work. Then, we outline our experience in introducing this work to the hundred odd participants that experienced the installation interactivity in Wellington, NZ. Finally, we share perspectives on the creation, conception and reception of this work in full.

### 5.1 Performer Perspective

To date, five different performers have embodied this role of the cyborg performer. The following represents the perspective of the first author who choreographed and has also performed the work. As choreographer, performer, and rehearsal director, her experience of the movement is arguably the richest and most nuanced; thus we select her perspective, which is only one of many, to share.

Performing Babyface is a constant oscillation between loving the way people admire me and hating the gaze through which they do so. I am a strong believer in the power of dance and choreography; so much of why I love dance and believe it’s powerful has to do with how it’s impressive. I lean into this heavily in the creation and performance of Babyface: the movement, and the frame that it’s housed in, are an impressive spectacle. There is a magical (if glitchy) connection between breath and motion; there is a motivation in catching and enjoying the syncing of a beat; there are flowy, complex sequences of motion, moments when I kick my legs high into the air, moments when the wings expand with my breathing in such a way, and I think to myself, “I’m doing something nobody else could think of, and few are able to execute.”

I’m deeply in tune with the structure that I’m wearing, and have a deeper awareness of the space immediately behind me. I can tell right away if something glitches or is wrong; I can feel the way the wings change the weight distribution on my back when they are extended or folded, I can hear and feel the vibration when the motor activates. I maintain a sense of control over how the wings move, as I am able to send my breath into the place where the sensor is situated. Familiarity and rehearsal have helped me understand the differences in how sensitive the sensor is in certain positions; for example, if my hands are high above my head, I need to make sure I’m sending breath deep into my ribcage, because when I lift my arms I have the tendency to puff out my chest and lessen the impact of my breath.

I’m especially aware of how much space I’m taking up, because it’s more space than I usually do. I have a generalized sensitivity to this as a woman and a former ballerina; I’m rather trained not to be a nuisance, to not take up too much space, and to be highly aware of how I’m occupying it. As the cyborg, my relationship to these tendencies changes. I take up much more space and I am highly noticeable; but I am extremely attentive to the positioning of my body and the speed at which I take certain motions.

The wings themselves walk this fine line between immovable and fragile; I don’t want to run into anything for fear of breaking the wings; however, the structure they are housed in limits motion through my upper back and shoulder girdle. This supports a more formal, upright posture, and distal motion in the hands and feet.

My experience of performing this work is sometimes so physically frustrating that the question, “do I need these wings” comes across my head. The answer: Yes. They have to be there. They have to move with me and separately from me, as a metaphor for expectation and control. They have to be ethereal and fragile and also a burdensome spectacle. They have to make me bigger, make me take up too much space, make visibly obvious the way I am often treated: as a pretty problem. They have to force me to manage space in this obnoxious way. And above all, the wings have to make me navigate the complicated relationship I’ve formed with them, so that people look at me and wonder what’s real and if I’m human, and then fully, deeply understand that I am.

### 5.2 Participant Perspectives

Participants entered the installation in a state of wanting to be entertained. Either they had come to the Arcade with this intent specifically or the installation caught their eye as they were walking by (although the majority of participants were in the former category). Many had just watched one of the hourly performances, but some had only seen the performer improvising in the space. In either case, they typically entered with some giggly trepidation: stepping up into the space^2^ that was often ringed by a collection of onlookers, they immediately recognized the experience of entering a performance area and being “onstage”.

Wrapping the sensor around the body of the participant also created a degree of transformation and role playing that created an understandable sense of self-consciousness and of being watched. Participants with friends onlooking would often laugh or joke with their friends in this moment. Others would look anxiously at the presenter and adjust their clothing nervously.

In both cases, during the “calibration”, participants focused on the instructions of the docent, seemingly in order to not “mess up” or “fail” at the task. This afforded an immediate shift from an external, presentational, and interactive mode of action to an internal, somatic, and meditative mode, as the participant listened to the docent and worked to make their bellies move with their breath as described by the facilitator.

Almost all participants had at least one “ah-ha” moment in controlling the wall. In a very few cases, we offered the ability to press with the hand when breath was not sufficient to create a differentiated enough threshold through belly deformation. From these first moments, as participants began to move around the space, forgetting their breath or twisting into poses and movements that deviated from an upright neutral in which the calibration took place, the wall unit would often fire in less predictable moments, with a fair amount of user-to-user variability.

Once this “ah-ha” moment occurred, the docent offered a choice: to move about the space, exploring on their own and interacting with the performer, or to move along to a track that would guide them through choreography. About half would elect to perform the choreography, which situated them inside the same performative frame as the professional dancer and gave onlookers a new perspective to many of the same choreographic structures, now on the body of this bystander.

When the participant elected to “perform the choreography” the performer would typically join in, helping the participant resolve the movement commands featured in the voiceover. The initial few commands are quite easy to buy into, e.g., “Look into the mirror” and “Put your hands behind your head”. Most participants followed these instructions with little vulnerability. But, then, when participants were asked to move their hips back-and-forth – as they faced away from the crowd – and the performer began a series of hip thrusts with a sexual, presentational tone – participants often began to feel embarrassed, silly, or even afraid. Some would even use physical strategies to tone down the protrusion of their hips from side-to-side.

Building from there, the audience, seeing the vulnerability and exposed nature of the participant role, was offered a new insight into the challenges of this cyborg character. What initially reads as a fun, disco-themed party becomes a creepy, awkward role to navigate. Thus, by the time the audience is, at the end of the experience, asked to “Clap for this beautiful angel” a range of reactions occurred. Some exuberantly joined in, laughing at their friend or companion as if they were attending a roast for the individual. Others felt uncomfortable at the request, worrying for the individual onstage. This awkwardness would be dissolved when the voiceover asked “Take a picture”, engaging everyone in a familiar activity: pose for the picture and post! This is a call and response that often happens in social settings where gender is performed. The performative simulation creates an opportunity to realize our own participation as audience members in the presentation, celebration, and limitation of feminine gender in society more broadly.

Throughout the performance, the framing of the wings as wide and flat, the opportunity for self-inspection and reflection in the mirrored fragments of the wings, the setup of the shipping container as pseudo-stage, and the motion of the body as posed, linear, and frontal make the whole experience highly “Instagrammable.” This ability and desire to be photographed becomes as an extension of seeking approval, likability and share-ability – a search for relevance via the archiving of self.

Indeed, the final prompt for viewers and participants alike is an invitation for the experience to be recorded: to take a picture of themselves augmented by an expressive machine that makes them, simultaneously, a magnificent and absurd spectacle. This photograph could be a point of pride: look at me, a part of this installation, in control, affecting this imposing structure with my own breath and motion. It could also be an embarrassing record of an uncomfortable, vulnerable, or objectifying experience. Regardless, this photograph adds to the existing archive of hundreds of millions of hyper-feminized images and photographs that fed the motion and material of this work to begin with.

### 5.3 Creative Perspective

*Babyface* is a work that seeks to meet audiences where they are. The motions, materials, structure, and interactive components of this work are meant to be familiar and immediately referential. We see a set of glittering wings on a wall, framing a hyper-femme cyborg barbie. We see our own image reflected back to us in mirrored elements that cast light around like a disassembled disco ball. We hear pop music beats and a feminine voice that reminds us of automated technology. We see poses that we’ve seen a hundred times before, on the bodies of “#cute” women on Instagram and across popular media.

*Babyface* is blunt with its spectacle as a pathway to its own subversion. An essential motivating question throughout our process was: how do we get audiences past the initial moment of, “oh my god, it’s a robot on stage!” and therefore able to engage with our higher level concepts? Our answer was to fully embrace this moment. If we can first confront audiences with a familiar, predictable stereotype (robot barbie with segmented motion and a fixed smile), we can then reveal the shortcomings of that stereotype (the struggle against aesthetic restriction and the vulnerability that comes with being on display). Indeed, many viewers of this work referred to it as “accessible,” noting the clarity of its narrative and immediacy of its references.

One feature of this accessibility is that kids found the installation really *fun*. While they are not our intended audience, it made the installation more popular, given that families could visit and enjoy with their children. To allow children, and many adults, to enjoy the work meant that some of our audience was missing out on the larger societal context and pointed critique embedded in the work.

In thinking about our adult audiences, “accessible” comes as its own kind of double-edged sword. Much in the same way that hyperbolic feminine performance can be viewed as cheap or simplistic, is “accessible,” as a point of feedback some kind of code for facile? Perhaps we as creators would not feel this way if we were not also women working at this murky intersection of arts and technology; if instead audiences asked us how it worked rather than if we knew how it worked; if they weren’t shocked when we said that we were designers, engineers, fabricators in addition to performers and choreographers.

We’re sure these inquiries were not intended with malice, more likely with interest and curiosity. However, like an adorable, curvy service bot or a voice assistant that defaults feminine, the assumptions are still there: you are woman, therefore one can assume you are capable of this, or not capable of that. So we look back into the glittering wall, at the shards of ourselves, fix our smiles and inhale, our wings sliding out to frame our experience in their limited expanse.

## 6 Discussion

This work alludes to broader principles that may apply to many robotics projects. Challenges around construction and design highlight distinct temporal cycles present in choreographing with live, intelligent bodies vs. building rigidly with units of programmed plastic. The need to express (and relative success of expressing) a particular meaning to audience members through both a passively-viewed performance and an actively-engaged experience highlights important context to consider when presenting robots. This section will highlight themes that may apply to future human-robot interaction and performing arts collaborations with different thematic aims and aesthetic textures.

• *Negotiating distinct design cycles.* Design thinking encourages iteration on many ideas, exploring the design space and improving intial ideas through refinement. A challenge when working with bespoke robotics in tandem with choreography is the distinct inertia of elements of the work. A piece of formed plastic, once manufactured, becomes a fixed design element. A piece of code is much more malleable but often takes significant debugging time to rework. A piece of movement can be adapted as late in the performance creation process as *onstage by a performer*. This can result in movement that befits the exact moment of a performance with a machine that looks extra or unneeded. Given the costs associated in creating the machine, this requires stringency in letting go of elements that are not needed. For example, we manufactured and carted over 100 additional plastic elements to New Zealand from the US that were unused. Instead, we spent our installation time refining the motion of the installed elements and the user experience in the container.

• *Accommodating the innate spectacle of robotic systems inside the established conventions of contemporary dance and performance art.* There is spectacle inherent in both robot and human bodies onstage. But, robots onstage have fewer precedent works and their novelty can get in the way of an authentic medium for expression. Moreover, robots are subject to wild hyperbole in their public presentations, including prior contemporary artists who have worked with the devices, portraying anthropomorphic devices augmented by theatrical and performative enhancements. In *Babyface*, we could not fully remove the spectacle of a woman controlling a robot onstage and instead leaned into that creating a character fueled by spectacle. The result was an inherent compromise between how the idea might have been communicated in a purely contemporary dance language of movement and how a utilitarian machine might be designed for efficient function.

• *Navigating being femme bodies who produce technically impressive work.* In the setting of the installation, audience members were often impressed with the scale and unseen functioning of the devices and their coordination with tightly performed movement vocabulary. This could elicit hyperbolic reactions that created two distractions from the main work. One, audience members may have been assigning more capability to the devices than they actually had. And, two, these reactions could prompt immediate questions that were often accompanied by an incredulity that was hard to navigate for the two petite female-presenting bodies presenting the work. For one, we worked hard to emphasize that the machine (led by our second author) would not exist without the artistic framework (led by our first author). Our goal was for the kind of collaboration where both engineering and arts skilled were valued equally. Indeed, the movement of the machine is a choreographic choice as well as an engineered design.

How these problems manifest in future work and other projects will always be different than how we encountered and handled them in *Babyface*. In fact, we leave this project with more questions than answers:

- *What is the correct balance in maintaining creative flexibility in machine and algorithm design so that the piece does not evolve beyond the machine, creating an unnecessary element?*
- *Where is the correct balance between explicating the inter-workings of the device and allowing for room for awe in the audience?*
- *How do we appropriately attribute the complexity of these collaborations?*

Striving for ways to accommodate design elements with various associated inertia and acknowledge the contribution of different kinds of knowledge is the only way to create novel human experiences with robots. Yet navigating two fields of distinct training and traditions requires some creativity and generosity on its own, separate from that required to create new work. In this spirit, these points and open questions present exciting avenues for future work, exploration, and experimentation.

## 7 Conclusion

*Babyface*, in its original kernel, was a short performance meant for a proscenium setting. This particular iteration of it took it out of a passive place and into an interactive space; in other words, what was once a performance meant for watching became a sculptural, robotic performance installation with multiple access points and channels for individual experience. It became a vehicle for novel play with emerging tools – for both performers and audiences. Situated in the artistic context created for *Babyface*, these tools become expressive and meaningful.

Further research into breath in the context of gender perfomativity, robotics, installation, and feminist studies will guide this project towards its next iteration. Future plans for this work include extending it into a stand-alone, evening length-experience, as well as fine tuning the interactive element. Technically speaking, the creators have plans for a multi-modal breath sensor, for both wearable and wall-mounted robotic structures. This would measure breath filling and emptying throughout the volume of the torso, accommodating nuances person-to-person in belly, breath, and chest breathing. Theatrically speaking, the creators would like to develop the interactive experience in a less open-ended setting, guiding audiences in a more controlled manner through the nuances of aesthetic awesomeness and physical limitation.

The work’s essential ideas around the spectacle of woman and machine, the pressure of feminine presentation inside of screen based media, and the limited view of femininity spread by technology provided the basis for an extension of this work into an interactive performance – one that seeks to make the experience of hyper-feminine spectacle literal and re-livable for its audience. These ideas became design parameters, extending to the materials and the construction of the performance space through to the motion of the machines and the humans wearing them. In our case, in exploiting the spectacle of robots and theatrical femininity, our work became an impactful form of social commentary. This particular end was unique to our goals but highlights some of the crucial steps to building expressive machines. The end affective goals must be part of the design process from concept to implementation, requiring expertise from both human-robot interaction and the performing arts.

## Data Availability

The original contributions presented in the study are included in the article/Supplementary Material, further inquiries can be directed to the corresponding author.

## Footnotes

1 The University of Illinois at Urbana-Champaign IRB provided a Not Human Subjects Research Determination (protocol #21203).

2 Note, for accessibility purposes, we could also trigger the wall from the sidewalk level, but we did not have any participants that needed to use that option.
